# Supplementary material for: Graph-based machine learning improves just-in-time defect prediction
Source: PLoS One. 2023 Apr 13;18(4):e0284077. doi: 10.1371/journal.pone.0284077 (PMC10101485; doi:10.1371/journal.pone.0284077)
Supplement: Attachment — Submitted filename: Rebuttal-Letter-PLOS-ONE.pdf [file pone.0284077.s001.pdf]

Response to Reviewer #1: We appreciate the Reviewer's comments. We believe that our responses will clarify all concerns raised by the Reviewer. We break down the Reviewer concerns into specific points (shown in italics) and then follow with our response. Changes in the manuscript have been highlighted in red.

- *The idea is interesting and has some novelty. The paper is clearly presented and easy to follow assuming the reader has some background knowledge.*

  Thank you for highlighting this. We are glad to hear that our work is novel and that readers will easily follow it.

- *Open science? Although a link is provided to the data (provided by another research group/Ning Li) there is no code or details for instance on how the holdout samples were undertaken. This means your analysis could not be reproduced. Please provide your python code etc.*

  Thanks for your suggestion. We have included pointers to the dataset and the Python code we used to allow the reproducibility of our results.

  Action: We have added the following pointers to the data and code near the end of the Introduction: "We are sharing the data [1] and code [2] used in this research so that our results can be reproduced."

- *There does seem a slight tendency to cherry pick results to "big up" their method. This isn't helpful or necessary. It would be better to either give the range of possible results and/or typical improvement.*

  Thanks for your suggestion. We have changed the way we present our results from absolute to relative differences to emphasize the true scale of differences between comparisons. In addition, we included results that use the Matthews correlation coefficient (MCC) to show the perspective from an unbiased classification metric.

  Action: We reworded our results when comparing to the baseline throughout the manuscript. In particular, they read as follows:

  "Abstract: We test our approach on 14 open-source projects and show that our best model can predict whether or not a code change will lead to a defect with an F1 score as high as 77.55% and a Matthews correlation coefficient (MCC) as high as 53.16%. This represents a 152% higher F1 score and a 3% higher MCC over the state-of-the-art JIT defect prediction."

  "Section 1: We show the potential of using this approach with higher classification results (i.e., with a 152% higher F1 score and a 3% higher MCC) compared to a recent benchmark on JIT defect prediction on early exposed defects over 14 large open-source projects [3]."

  "Section 1: Our results show that the graph-based approach provides a 152% higher F1 score and a 3% higher MCC than the state-of-the-art."

  "Section 4.1: Finally, the MCC scores obtained with the proposed framework are higher than the baseline for random forest and XGBoost classifiers (i.e., 3% [from 51.41% to 53.16%] and 2% [from 51.41% to 52.34%] in Setting 1 and 1% [from 51.41% to 52.15%] and 2% [from 51.41% to 52.34%] in Setting 2)."

  "Section 4.2: We also compare the performance of different classifiers in each

setting by using the mean of AUC-PR. Under Setting 1, the three classifiers perform similarly with a slight advantage of logistic regression (80.74%) over random forest (80.22%) and XGBoost (79.93%). The relative performance increase of logistic regression based on AUC-PR is at most 1% over XGBoost. Note, however, that the XGBoost classifier tends to perform best in terms of average ranking across the 14 repos (i.e., [1.64] over random forest [2.07] and logistic regression [2.29]). Under Setting 2, the best performing classifier based on average AUC-PR is logistic regression (81.51%) followed by XGBoost (80.61%) with a relative performance increase of at most 1%. However, like in Setting 1, the best performing classifier based on rankings is again XGBoost (1.86) over logistic regression (1.93) and random forest (2.21). This suggests that across repos, XGBoost consistently has the best performance despite outliers."

"Section 5: We showed that the relative performance increase in the JIT defect prediction task is 152% for F1 score and 3% for MCC over the state-of-the-art baseline when using Setting 1."

"Section 6: The proposed framework outperforms the state-of-the-art in JIT defect prediction with a higher average F1 score (152%) and higher average MCC (3%) across 14 open-source projects."

- *I'm concerned about the classification performance evaluation. The authors note the problems relating to imbalanced datasets and for that reason advocate AUC-PR (fair enough) but then extensively quote F1 performance stats which seems odd (e.g., l438).*

  We used F1 scores to report the classification results of our proposed framework because it allows direct comparison with the results provided by the baseline work [3]. Note that the baseline work does not report AUC-PR scores, so we do not emphasize that in the manuscript as we would have liked.

- *Another problem with AUC style stats is they are based on entire families of classifier (many of which are uninteresting in a practical sense) so unless classifier x strictly dominates y knowing $AUC_x > AUC_y$ is not informative. There are also other concerns (see [1-2]).*

  Thank you very much for pointing this out. We reviewed the references you suggested as well as additional related work in the area such as [4, 5] and understand the AUC-style metrics limitations. We agree that AUC-style metrics refer to a family of possible classifiers at different thresholds instead of a specific classifier. Because in practice only a single classifier can be deployed, we cannot state that one classifier is better than another unless the ROC curve of one of them strictly dominates the ROC of the other.

  Action: We added a clarification about the use of AUC-style metrics for comparing classifiers in Section 5. In particular, it reads:
  "Use of AUC-Style Metrics to Compare Classifiers. Note that AUC-style metrics are based on a family of possible classifiers at different thresholds instead of a specific classifier. Because in practice only a single classifier can be deployed, we also include other metrics that focus on a single threshold (i.e., F1 score and MCC) to better qualify the best performing classifier."

- *I'd consider using Matthews correlation coefficient/phi or Youden's J (which compares against a guessing strategy).*

Thank you for your suggestion. We have expanded our results using the Matthews correlation coefficient (MCC) which compares against a guessing strategy.

Action: We have reported additional results, including MCC and updated our manuscript accordingly. In doing so, we have updated the following sections with the inclusion of MCC. In particular, they read as follows:

"Section 3.5: We used confusion matrix–based metrics to compute the performance of the proposed methods, which have been widely employed for JIT defect prediction [6, 3]. The basis for comparison is counting the number of code changes that were labeled as true positives (TP), false positives (FP), false negatives (FN), and true negatives (TN). We focus on *Precision*, defined as $\frac{TP}{TP+FP}$, which gives the likelihood that a detected change is defect-prone; *Recall*, defined as $\frac{TP}{TP+FN}$, which gives the likelihood that a defect-prone change is detected; *F1 score*, defined as $2 \times \frac{precision \times recall}{precision + recall}$, which combines precision and recall to provide a balanced view between them; and the Matthews correlation coefficient (MCC), defined as $\frac{TP \times TN - FP \times FN}{\sqrt{(TP+FP) \times (TP+FN) \times (TN+FP) \times (TN+FN)}}$, which is the Pearson correlation for a contingency table [7]. MCC takes values in the range $[-1, 1]$, with extreme values -1 and $+1$ reached in the case of perfect misclassification and perfect classification, respectively. MCC equals 0 is equivalent to the expected value for the coin-tossing classifier. We include the MCC metric because the above metrics overemphasize the positive class while not putting much emphasis on the negative class, which is also important for defect prediction. Note, however, that among the 14 repos that we analyzed, they have different levels of imbalance with only one of them (HBase in Table 2) showing extreme imbalance (i.e., less than 1% for the minority class [8]). Therefore, we report results by using precision, recall, and F1 scores in addition to MCC. We let the reader explore the literature [4, 5] for a more thorough discussion of the advantages of MCC over other classification metrics. We evaluate the performance of our proposed framework against a state-of-the-art baseline that uses software-level characteristics as features of a random forest classifier [3]."

"Section 4.1: We applied the two settings of graph-based ML classifiers and measured the performance in the classification task by using the mean and average ranks of Precision, Recall, F1 score, AUC-PR, and MCC across the 14 repositories of code. These metrics are defined in Section 3.5. Table 1 summarizes the results based on the performance evaluation in the two settings (see Section 3.4 for details). We observe that the graph-based ML classifiers based on random forest and XGBoost (in both settings) outperform the baseline that uses a random forest classifier in terms of F1 score and MCC."

"Section 4.1: Finally, the MCC scores obtained with the proposed framework are relatively higher than the baseline for random forest and XGBoost classifiers (i.e., 3% [from 51.41% to 53.16%] and 2% [from 51.41% to 52.34%] in Setting 1 and 1% [from 51.41% to 52.15%] and 2% [from 51.41% to 52.34%] in Setting 2). These MCC scores reflect a moderate positive relationship (between 0.3 and 0.7 based on [9]). Yet, this does not hold for the logistic regression classifier. Note, however, that the F1 score and MCC results are concordant when using random forest and XGBoost classifiers, meaning that we obtain consistent preferred classifiers regardless of whether we use an F1 score or MCC score when comparing with the baseline [5]. This provides empirical evidence that the proposed frame-

work performs better than the state-of-the-art baseline when using classifiers of similar complexity."

		Precision		Recall		F1 score		MCC		AUC-PR	
		Mean	Avg. R	Mean	Avg. R	Mean	Avg. R	Mean	Avg. R	Mean	Avg. R
Setting 1	Logistic Regression	0.6050	2.57	0.6005	2.64	0.5871	2.86	0.3393	2.64	**0.8074**	2.29
	Random Forest	**0.7359**	**2.00**	**0.8241**	**1.86**	**0.7724**	**1.79**	**0.5316**	**1.86**	**0.8022**	**2.07**
	XGBoost	**0.7341**	**1.43**	**0.8239**	**1.50**	**0.7755**	**1.36**	**0.5234**	**1.50**	0.7993	**1.64**
Setting 2	Logistic Regression	0.6407	2.50	0.7773	2.36	0.6950	2.36	0.3231	2.64	**0.8151**	**1.93**
	Random Forest	**0.7343**	**2.07**	**0.8237**	**2.00**	**0.7714**	**2.00**	**0.5215**	**2.07**	0.8015	2.21
	XGBoost	**0.7418**	**1.43**	**0.8235**	**1.64**	**0.7748**	**1.64**	**0.5234**	**1.29**	**0.8061**	**1.86**
Beseline [3]	Random Forest	0.4673	1.03	0.7644	1.03	0.3083	1.03	0.5141	1.03	—	—

Table 1: **Classification results for both settings with the two best performing classifiers shown in bold text.**

- *Lack of tuning may be misleading (l480-2). I appreciate you've not tuned any method but it seems a bit unrealistic.*

  Thank you for pointing out that our statement could be understood in this way. We did not mean that we used default [hyper]parameter values for the ML classifiers but only for the graph algorithms computed from the contribution graphs that use the Neo4j Graph Data Science API (Settings 1 and 2). The documentation for the graph algorithm parameters can be found in [10]. We have clarified this issue when describing the limitations of our work to highlight that we used the default parameters only in the graph algorithms. In fact, we performed a grid search over some of the hyperparameters of the ML classifiers that we compared to achieve better results. We detailed this activity in Section 3.4.

  Action: We have clarified this when discussing the limitations of our work in Section 5. In particular, it reads:
  "**Use of Default Graph Algorithms' Parameter Values.** We run the graph algorithms to extract features from the one-mode projection graphs by using their default parameters. See related documentation of graph algorithm parameters in [10]. Thus, we do not optimize results by tuning these parameters."

- *Abstract (and l486): by as much as 46.72% but this isn't the representative case. I think the authors can reframe this claim in a more reasonable way without losing the impact or value of their work. We're not in the advertising business ;-)*

  As per your suggestion above (page 1), we have reworded the results we obtained when comparing with the baseline. In particular, we changed the way we presented our results from absolute to relative differences to emphasize the true scale of differences between comparisons. In addition, we included results that use the Matthews correlation coefficient (MCC) to show the perspective from an unbiased classification metric. Please refer to the specifics changes starting in page 1 of this rebuttal letter.

- *l398: lower −> fewer (being a bit pedantic here!)*

  Thanks for your suggestion. We have changed it in the updated version of the manuscript.

  Action: We have changed that in the manuscript. In particular, it reads: "This means that the proposed framework produces fewer FPs, even when using a simpler classifier, such as logistic regression."

We would like to thank the Reviewer again for taking the time to review our manuscript.

Response to Reviewer #2: We appreciate the Reviewer's comments. We are glad to hear that our work is "very important" providing "sufficient introduction and research motivation" with methods that are "new and innovative." We believe that our responses will clarify all concerns raised by the Reviewer. We break down the Reviewer concerns into specific points (shown in italics) and then follow with our response. Changes in the manuscript have been highlighted in red.

1. *The paper should have more case studies related to work.*

   We agree that having results on more case studies would be interesting. Our study focuses on an extensive empirical investigation that uses a dataset comprising 14 out of 18 well-known, open-source multi-application projects coded in Java (17) and C++ (1). This makes our case study as comprehensive as possible (see more details in Section 3.2). Please note that our case study is bigger and more diverse than the datasets used in recent published papers in this area: [11] focuses on six open-source and five commercial projects, [12] focuses on six open-source projects, [13] focuses on two open-source projects, [14] focuses on six open-source projects, [15] focuses on six open-source projects, and [16] focuses on ten open-source projects. For that reason, we believe that our results will provide a useful baseline for further research in this area and this is supported by data [3] and code [2]. We, however, have included your point as a consideration for future study. Thank you for your suggestion.

   Action: We have added the following to the Conclusion section: "In addition, future work could include benchmarking the effectiveness of the proposed framework with more diverse codebases to further improve the generalizability of the proposed method."

2. *The notations should have a particular table.*

   Thanks for pointing this out. We have included a Table with the notation used in our work.

   Action: We have added the following Table at the end of Section 3.1. The table details the notation we used: "We summarize the notation used to model the contribution graphs and their extracted features in each Setting in Table 2.

Context	Notation	Description
Contribution graph	$\mathcal{H}_\top$	The set of top nodes or developers
	$\mathcal{H}_\bot$	The set of bottom nodes or files
	$\mathcal{V}$	The set of contribution graph nodes
	$\mathcal{W}$	The incident matrix of weights
	$\mathcal{G} = (\mathcal{V}, \mathcal{W})$	The weighted bipartite graph capturing software changes
	$\mathcal{G}_\top$	The developer-based one-mode projection
	$\mathcal{W}_\top$	Weighted matrix in the developer-based one-mode projection
	$\mathcal{W}_\top^\ell$	The set of labeled $\mathcal{W}_\top$ edges
Setting 1	$d(i)$	The degree of node i
	$b(i)$	The betweenness of node i
	$c(i)$	The closeness of node i
	$h(i)$	The harmonic centrality of node i
	$PR(i)$	The PageRank of node i
Setting 2	C	The community partition
	f	The node embedding mapping function

Table 2: **Summary of notation used.**

3. *The time complexity of each method must be defined in the paper.*

Thank you for pointing this out. We have defined the time complexity of each of the ML classifiers that we contrast in our study.

Action: We have added the following to Sec. 3.4: "Each classifier is described below along with their train and test time complexities. Here, n refers to the number of samples, m refers to the number of features, t is the number of trees, d is the height of the trees, and x is the number of non-missing entries in the training dataset [17];" Section 3.4.1: "The training and testing time complexities of logistic regression are $O(nm)$ and $O(m)$, respectively;" Section 3.4.2: "The training and testing time complexities of random forest are $O(tn \log nm)$ and $O(mt)$, respectively;" and Section 3.4.3: "The training and testing time complexities of XGBoost are $O(tdx \log n)$ and $O(td)$, respectively."

4. *Literature work should have a table in which each paper's method, problem, constraints, and research should be analyzed in detail in tabular form.*

Thanks for pointing this out. We have expanded our literature review discussion and included a table to contrast related work in the area.

Action: We have added the following at the end of Section 2.3 to detail close related work: "An important aspect of JIT defect prediction is identifying and extracting the independent variables used to build JIT defect prediction models. Generally, there are two approaches for doing that: feature engineering and feature learning. In the former, features are designed manually. In the latter, features are learned automatically via algorithms as feature representations from the data.

Early work on JIT defect prediction through feature engineering focused on using features derived from software change metrics as those directly computed from code changes. They include variables related to diffusion (e.g., number of modified subsystems), size (e.g., lines of code added), history (e.g., number of developers who modified the files), and experience (e.g., developer experience) [18]. The origin of JIT defect prediction through feature engineering is usually attributed to Mockus and Weiss [19]. Working with a large, closed telecommunication code base, Mockus and Weiss introduced the idea of quantifying software change properties to predict defects for initial maintenance requests (IMRs) when the IMRs consist of multiple changes. Kim et al. [20] focused on predicting individual defect-prone changes and applied this method to a variety of open-source code bases. Kamei et al. [11] extended previous work by applying it to open-source and commercial code bases across multiple industries. Jiang et al. [21] introduced a personalized defect prediction approach by building a separate prediction model for each developer. Kononenko et al. [22] added features extracted from code review databases, thereby leading to an increase in the explanatory power of JIT defect prediction models. Kamei et al. [23] evaluated the performance of JIT defect prediction in projects still in their initial development phases. In doing so, they showed that JIT models trained using data from projects with sufficient history are viable candidates for JIT defect prediction in projects with limited historical data.

Early work on JIT defect prediction via feature learning focused on estimating feature representations by extracting the amount of information in the commit message. Deep learning approaches, and deep neural networks in particular, are predominantly used to learn feature representations for JIT defect prediction. For example, Yang at al. [12] built a set of expressive features from a set of initial changes by leveraging a deep belief network algorithm. Later, they trained

an ML classifier on selected features. Barnett et al. [24] mined commit message content by using SpamBayes classifiers [25] as filters. More recently, Hoang et al. [13] proposed an end-to-end deep learning framework, named DeepJIT, which automatically extracts features from code changes and commit messages and uses them to classify defects. Xu et al. [26] proposed a cross-triplet deep feature embedding method, called CDFE, for cross-app JIT defect prediction in mobile apps. The CDFE method incorporates a cross-triplet loss function into a deep neural network to learn high-level feature representation for the cross-app data. This loss function shortens the distance of commits with similar labels while lengthening the distance between commits with different labels. Zhuang et al. [16] proposed a method to represent code semantics based on Abstract Syntax Tress (ASTs). This method works by comparing the AST of source code before and after a change for extracting change sequences that are then mapped to a numeric vector by using word-embedding models.
Table 3 summarizes closely related previous work on JIT defect prediction that uses feature learning. For a comprehensive survey on JIT defect prediction, we refer the reader to the work by Zhao et al. [18].
To the best of our knowledge, apart from features derived through feature engineering (based on software code change metrics) and features derived using feature learning, no prior studies have investigated the use of features derived from the contribution graphs to inform graph-based ML classifiers to predict the risk of introducing defect-prone changes."

Study	Year	Primary Topic	Constraint
Yang et al. [12]	2015	Proposed Deeper, which consists of a deep belief network and a logistic regression classifier for JIT defect prediction	Deeper does not fully exploit the true benefits of deep learning because it uses the same set of traditional features derived from feature engineering.
Barnett et al. [24]	2016	Investigated the benefits of adding commit message features for JIT defect prediction	Leveraging commit message proneness to be defective by using a SpamBayes classifier may produce overly specific scores for systems to which they will be applied.
Hoang et al. [13]	2019	Introduced DeepJIT, an end-to-end JIT defect prediction model that learns feature representations from tokenized software changes and commit messages	Because code is written using an open, rapidly changing vocabulary, word embeddings may introduce noise and then cause a negative impact on the performance of the cross-project defect prediction.
Xu et al. [26]	2021	Designed CDFE, a deep neural network with triplet loss function for cross-project JIT defect prediction in mobile apps	Using labeled commit data from other mobile apps to assist the labeling of the mobile app at hand may be impacted by the imbalanced nature of JIT defect prediction.
Zhuang et al. [16]	2022	Introduced ACE based on Abstract Syntax Trees (ASTs) by comparing the AST of source code before and after a change and computing change sequences that are mapped to word embeddings	Experiments conducted in projects coded on Java. AST nodes of different programming languages may differ, thereby influencing JIT defect prediction performance.

Table 3: **Summary of JIT defect prediction works by using feature learning.**

5. *The research findings and limitations must be defined before the conclusion of the work.*

   We discussed research findings and limitations in the Discussion section before the conclusion of the paper. Please refer to Section 5.

We would like to thank the Reviewer again for taking the time to review our manuscript.

Response to Reviewer #3: We appreciate the Reviewer's comments. We believe that our responses will clarify all concerns raised by the Reviewer. We break down the Reviewer concerns into specific points (shown in italics) and then follow with our response. Changes in the manuscript have been highlighted in red.

- *Authors mentioned "The core of our contribution is problem formulation. In particular, we leverage contribution graphs to extract graph-related features that inform classification models when classifying defect-prone changes." I do not understand if the contribution is more on data classification modeling or proposing new feature vector OR new framework. Elaborate.*

  Thank you for your observation. We have clarified the main contribution of our work in the Introduction. We want the reader easily extract our main methodological innovation that leads to better classification results.

  Action: We have clarified our main methodological innovation. In particular, it reads: "The core of our contribution is data modeling by using contribution graphs. In particular, we leverage contribution graphs as a modeling framework to capture the changes that developers make to software files. We used this abstraction as the basis of edge classification. Specifically, from the contribution graphs, we extract graph-related features that inform edge classification models when classifying defect-prone changes."

- *Reorganize the methodology section into phases with one main diagram that represent[s] each phase as well as each step into distinct phase.*

  Thanks for your suggestion. Following your recommendation, we have included a diagram that represents each phase in the methodology as well as how each step maps into distinct phases in Section 3.

  Action: We include Fig. 1 to distinguish between phases and matched their corresponding steps in our proposed method. In particular, it reads: "An overview of the proposed framework is presented in Fig 1. Our method is composed of three main phases: dataset generation, training, and testing. In the dataset generation phase, we build a labeled commit dataset by combining the extracted graph features from the contribution graph (see Section 3.1) and bug-inducing commits produced by the SZZ algorithm. During the training phase, we process the training data (see Section 3.3) by conducting data preparation (see Section 3.3.1), model training (see Section 3.3.2), and selection (see Section 3.3.3) on a subset of classifiers (see Section 3.4). During the testing phase, we prepare the data from given code changes and feed it into the trained model (see Section 3.3.4). Predictions from the classifiers are used to estimate the quality of the predictions (see Section 3.5). We associated each step in the proposed method (and their subsequent section numbers) with the corresponding phase in Fig 1."

- *Figure 1 and 2 are missing.*

  We uploaded image files separately from the manuscript submission following PLOS ONE policy. The individual image files that we uploaded separately will be automatically included in the reviewers PDF.

- *Expand the comparison with state of art work (at least 3–4 works) not only work [[3]].*

  We agree that benchmarking the results of the proposed framework with other state-of-the-art methods beyond [3] would be an interesting to exploration. Our

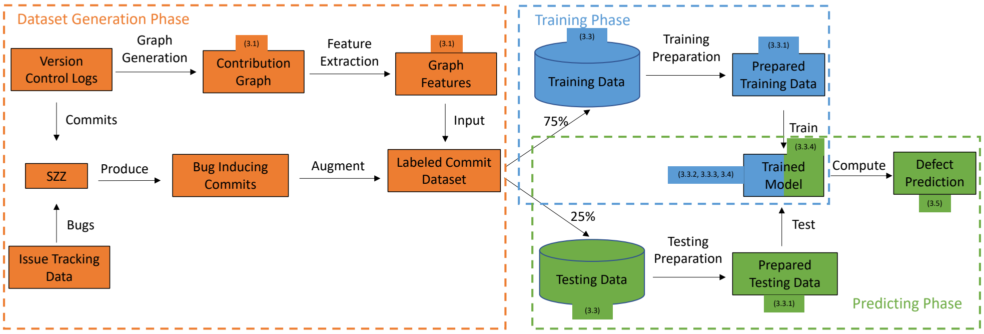

Figure 1: **Graph-based ML JIT defect prediction pipeline.**

experimental study focuses on the dataset [1] made available by [3], which includes labeled defect introduction fix pairs on 18 well-known, open-source multi-application projects coded in Java (17) and C++ (1). This makes our case study as comprehensive as possible. See more details in Section 3.2. Therefore, comparing their results is the natural choice. In addition, we studied the references from [3] and did not find additional research that reported classification results for JIT defect prediction using this dataset.

Please note that our case study is bigger and more diverse than the datasets used in other published papers in this area: [11] focused on six open-source and five commercial projects, [12] focused on six open-source projects, [13] focused on two open-source projects, [14] focused on six open-source projects, [15] focused on six open-source projects, and [16] focused on ten open-source projects. For that reason, we believe that our results provide a comprehensive baseline for further research in this area and is supported by data [1] and code [2]. We, however, have included your point as a consideration for future study. Thank you for your suggestion.

Action: We have added the following to the Conclusion section: "In addition, future work could include benchmarking the effectiveness of the proposed framework with more diverse codebases to further improve the generalizability of the proposed method."

We would like to thank the Reviewer again for taking the time to review our manuscript.

References

[1] Ning L. JiTReliability; 2020. `https://github.com/lining-nwpu/JiTReliability.git`.

[2] Bryan J. defect-prediction; 2022. `https://github.com/jtbryan/defect-prediction`.

[3] Tian Y, Li N, Tian J, Zheng W. How Well Just-In-Time Defect Prediction Techniques Enhance Software Reliability? In: QRS; 2020.

[4] Chicco D, Jurman G. The advantages of the Matthews correlation coefficient (MCC) over F1 score and accuracy in binary classification evaluation. BMC genomics. 2020;21(1):1–13.

[5] Yao J, Shepperd M. The impact of using biased performance metrics on software defect prediction research. Information and Software Technology. 2021;139:106664.

[6] Liu J, Zhou Y, Yang Y, Lu H, Xu B. Code churn: A neglected metric in effort-aware just-in-time defect prediction. In: ESEM; 2017.

[7] Powers DM. Evaluation: from precision, recall and F-measure to ROC, informedness, markedness and correlation. arXiv preprint arXiv:201016061. 2020;.

[8] Google. Imbalanced Data; 2022. `https://developers.google.com/machine-learning/data-prep/construct/sampling-splitting/imbalanced-data`.

[9] Ratner B. The correlation coefficient: Its values range between+ 1/- 1, or do they? Journal of targeting, measurement and analysis for marketing. 2009;17(2):139–142.

[10] Neo4j. Graph algorithms; 2022. `https://neo4j.com/docs/graph-data-science/current/algorithms/`.

[11] Kamei Y, Shihab E, Adams B, Hassan AE, Mockus A, Sinha A, et al. A large-scale empirical study of just-in-time quality assurance. IEEE Trans Softw Eng. 2012;39(6):757–773.

[12] Yang X, Lo D, Xia X, Zhang Y, Sun J. Deep learning for just-in-time defect prediction. In: QRS; 2015.

[13] Hoang T, Dam HK, Kamei Y, Lo D, Ubayashi N. DeepJIT: an end-to-end deep learning framework for just-in-time defect prediction. In: MSR; 2019.

[14] Qiao L, Wang Y. Effort-aware and just-in-time defect prediction with neural network. PloS one. 2019;14(2):e0211359.

[15] Kondo M, German DM, Mizuno O, Choi EH. The impact of context metrics on just-in-time defect prediction. Empir Softw Eng. 2020;25(1):890–939.

[16] Zhuang W, Wang H, Zhang X. Just-in-time defect prediction based on AST change embedding. Knowl Based Syst. 2022;248:108852.

[17] Surana S. Computational Complexity of Machine Learning Models - II; 2021. `https://www.kaggle.com/general/263127`.

[18] Zhao Y, Damevski K, Chen H. A Systematic Survey of Just-In-Time Software Defect Prediction: Online Supplement. ACM Computing Surveys. 2022;.

[19] Mockus A, Weiss DM. Predicting risk of software changes. Bell Labs Tech J. 2000;5(2):169–180.

[20] Kim S, Whitehead EJ, Zhang Y. Classifying software changes: Clean or buggy? IEEE Trans Softw Eng. 2008;34(2):181–196.

[21] Jiang T, Tan L, Kim S. Personalized defect prediction. In: ASE; 2013.

[22] Kononenko O, Baysal O, Guerrouj L, Cao Y, Godfrey MW. Investigating code review quality: Do people and participation matter? In: ICSME; 2015.

[23] Kamei Y, Fukushima T, McIntosh S, Yamashita K, Ubayashi N, Hassan AE. Studying just-in-time defect prediction using cross-project models. Empir Softw Eng. 2016;21(5):2072–2106.

[24] Barnett JG, Gathuru CK, Soldano LS, McIntosh S. The relationship between commit message detail and defect proneness in java projects on github. In: 2016 IEEE/ACM 13th Working Conference on Mining Software Repositories (MSR). IEEE; 2016. p. 496–499.

[25] Meyer TA, Whateley B. SpamBayes: Effective open-source, Bayesian based, email classification system. In: Proc. of the 7th Annual Collaboration, Electronic Messaging, Anti-Abuse and Spam Conference. Citeseer; 2004.

[26] Xu Z, Zhao K, Zhang T, Fu C, Yan M, Xie Z, et al. Effort-aware just-in-time bug prediction for mobile apps via cross-triplet deep feature embedding. IEEE Transactions on Reliability. 2021;71(1):204–220.
